# Quantification of Cable Bacteria in Marine Sediments via qPCR

**DOI:** 10.3389/fmicb.2020.01506

**Published:** 2020-07-03

**Authors:** Jeanine S. Geelhoed, Sebastiaan J. van de Velde, Filip J. R. Meysman

**Affiliations:** ^1^Department of Biology, University of Antwerp, Antwerp, Belgium; ^2^Department of Earth and Planetary Sciences, University of California, Riverside, Riverside, CA, United States; ^3^Department of Biotechnology, Delft University of Technology, Delft, Netherlands

**Keywords:** cable bacteria, quantitative PCR, *Desulfobulbaceae*, marine sediment, amplicon sequencing, current density, oxygen consumption rate

## Abstract

Cable bacteria (Deltaproteobacteria, *Desulfobulbaceae*) are long filamentous sulfur-oxidizing bacteria that generate long-distance electric currents running through the bacterial filaments. This way, they couple the oxidation of sulfide in deeper sediment layers to the reduction of oxygen or nitrate near the sediment-water interface. Cable bacteria are found in a wide range of aquatic sediments, but an accurate procedure to assess their abundance is lacking. We developed a qPCR approach that quantifies cable bacteria in relation to other bacteria within the family *Desulfobulbaceae*. Primer sets targeting cable bacteria, *Desulfobulbaceae* and the total bacterial community were applied in qPCR with DNA extracted from marine sediment incubations. Amplicon sequencing of the 16S rRNA gene V4 region confirmed that cable bacteria were accurately enumerated by qPCR, and suggested novel diversity of cable bacteria. The conjoint quantification of current densities and cell densities revealed that individual filaments carry a mean current of ∼110 pA and have a cell specific oxygen consumption rate of 69 fmol O_2_ cell^–1^ day^–1^. Overall, the qPCR method enables a better quantitative assessment of cable bacteria abundance, providing new metabolic insights at filament and cell level, and improving our understanding of the microbial ecology of electrogenic sediments.

## Introduction

Cable bacteria are long, multicellular, filamentous bacteria that transport electrons from cell-to-cell along the filament over distances of up to several centimeters (Pfeffer et al., 2012; Bjerg et al., 2018; Meysman, 2018; Meysman et al., 2019). This long-distance electron transport enables an electrical coupling of sulfide oxidation in deeper anoxic sediment layers to oxygen reduction near the sediment surface (Nielsen et al., 2010). Electrogenic sulfur oxidation (e-SOx) by cable bacteria makes an important contribution to sulfur cycling in aquatic sediments (Risgaard-Petersen et al., 2012; Malkin et al., 2014; Burdorf et al., 2017; Sandfeld et al., 2020), and has a strong imprint on the local geochemistry of the seafloor (Risgaard-Petersen et al., 2012; Meysman et al., 2015). The sulfide oxidizing half-reaction results in an acidification of the pore water, leading to the dissolution of minerals, such as carbonates and sulfides, thereby greatly affecting the cycling of elements like Ca, Fe, Mn, P and trace elements (Risgaard-Petersen et al., 2012; Rao et al., 2016; Sulu-Gambari et al., 2016; van de Velde et al., 2016, 2017). The dissolution of iron sulfide supplies additional sulfide to the cable bacteria and results in a strong increase of the ferrous iron (Fe^2+^) concentration in the pore water. Part of this aqueous Fe^2+^ will diffuse to the oxic sediment surface, where it will reoxidize and reprecipitate as iron(III) (hydr)oxides (Seitaj et al., 2015; Rao et al., 2016). This precipitated iron(III) (hydr)oxide plays a critical role in coastal ecosystems experiencing seasonal hypoxia, because it intercepts sulfide that is released from the sediment to the bottom water, and hence reduces the risk of sulfide toxicity to fauna in seasonally stratified coastal systems (Seitaj et al., 2015). Accordingly, given this strong environmental impact of cable bacteria, it is adamant to accurately quantify both their abundance and activity in aquatic sediments.

Phylogenetic studies of the 16S rRNA gene of cable bacteria show affiliation to the family *Desulfobulbaceae*, in which cable bacteria form a sister clade of the genus *Desulfobulbus* (Pfeffer et al., 2012; Trojan et al., 2016; Kjeldsen et al., 2019). Two genera of cable bacteria have been identified in aquatic surface sediments. The genus *Candidatus* Electrothrix contains sequences originating from marine and salt marsh sites and comprises four species, *Ca.* Electrothrix aarhusiensis, E. communis, E. japonicus and E. marina. The genus *Candidatus* Electronema encompasses sequences of cable bacteria from freshwater sediments and comprises two species, *Ca.* Electronema nielsenii and En. palustris (Trojan et al., 2016). Cable bacteria have also been reported from a groundwater aquifer. These subsurface sequences were also affiliated to the *Desulfobulbaceae*, but clustered more closely with *Desulfurivibrio alkaliphilus* with 91% sequence identity (Müller et al., 2016, 2019). Currently, no cable bacteria isolates are available in pure culture.

The natural occurrence of cable bacteria has been documented in sediments from a range of marine and freshwater environments (Malkin et al., 2014; Risgaard-Petersen et al., 2015; Burdorf et al., 2017). However, the actual abundance of cable bacteria has only been quantified in a few locations. Quantification of cable bacteria abundance has thus far been performed using fluorescence *in situ* hybridization (FISH), and the DSB706 probe is most commonly used to quantify cable bacteria densities (Pfeffer et al., 2012; Malkin et al., 2014, 2017; Schauer et al., 2014; Risgaard-Petersen et al., 2015; Vasquez-Cardenas et al., 2015; Burdorf et al., 2016; van de Velde et al., 2016; Hermans et al., 2019). The DSB706 probe targets most members of the family *Desulfobulbaceae* and the genus *Thermodesulforhabdus* (Loy et al., 2002), and therefore, only the hybridized filaments and not the single cells are counted as cable bacteria. The ELF645 probe has been developed to more narrowly target the monophyletic clade of surface sediment cable bacteria (Pfeffer et al., 2012; Marzocchi et al., 2014), while additional species-level probes are targeting specific species within the *Ca.* Electrothrix and *Ca.* Electronema genera (Trojan et al., 2016; Marzocchi et al., 2018).

The quantification of cable bacteria by FISH has been shown to suffer from substantial variation, leading to standard errors of 50–80% when using three replicates (Risgaard-Petersen et al., 2015). This variation is likely caused by the small volume of sediment that is used in sample preparation to avoid high background of sediment particles and non-hybridized cells. Typically, only ∼1 μl of wet sediment is deposited onto a filter of 25 mm diameter, and this small amount of sample allows for strong variability in the FISH analysis between samples. An alternative approach for quantification is the use of qPCR using primers targeting the 16S rRNA gene of specific phyla or targeting specific functional genes (Agogue et al., 2008; Kato et al., 2009; Miyashita et al., 2009; Blazejak and Schippers, 2011; Pohlner et al., 2017).

Here we explore the use of qPCR as a method to quantify the abundance of cable bacteria in marine and salt marsh sediments. We used primers that are selective either for cable bacteria of the genus *Ca.* Electrothrix or for the larger *Desulfobulbaceae* family. We enumerated cable bacteria, *Desulfobulbaceae* and total bacteria in sediments from laboratory sediment incubations with and without the presence of cable bacteria. Also, we compared the qPCR results with abundance estimates obtained using 16S rRNA amplicon sequencing. Filament and cell densities of cable bacteria are subsequently combined with electrical current densities derived from a detailed assessment of the sediment geochemistry. This allows to estimate the electron flow and metabolic activity at the single filament and single cell level.

## Materials and Methods

### Sediment Collection and Laboratory Sediment Incubations

Sediment for laboratory incubations was collected from Lake Grevelingen (Netherlands), a coastal basin in which the presence of cable bacteria has been extensively documented (e.g., Malkin et al., 2014; Seitaj et al., 2015). The sampling site (Den Osse basin, site S1, 51.747°N, 3.890°E, salinity 33, water temperature 11.8°C) is at 34 m water depth and sediment was taken using gravity cores (60 mm inner diameter, 60 cm long; Uwitec, Austria).

Sediment was sieved (<500 μm) and homogenized, left to settle overnight and repacked into plexiglass core liners of 40 mm diameter with the sediment surface level at the top of the core liner. The sediment cores were incubated at a temperature of 16°C in containers containing artificial seawater at salinity 30 (Instant Ocean Sea Salt). The containers were kept dark to prevent photosynthesis and the overlying water was continuously aerated. Four cores were incubated in a container that only contained freshly prepared artificial seawater (denoted “unamended incubation”). In a second treatment (denoted the “stimulated incubation”), four cores were incubated in a container that already contained “inoculum cores” with active cable bacteria enrichments that were started 32 days earlier (Supplementary Figure S1).

### Microsensor Profiling

Pore water concentration depth profiles of free sulfide (H_2_S), oxygen (O_2_) and pH were recorded using commercially available micro-electrodes (Unisense, Denmark) after 22 days of incubation. A single depth profile of H_2_S and pH, and duplicate profiles of O_2_, were taken in 2 cores from each treatment following the procedure outlined in van de Velde et al. (2016). Sensors were calibrated following the standard calibration procedures as described previously (Malkin et al., 2014) (H_2_S: 5 point standard curve using Na_2_S standards; O_2_: 2 point calibration using 100% in air bubbled seawater and the anoxic zone of the sediment; pH: 3 NBS standards (pH = 4, 7, 10) and TRIS buffer). The pH data are reported on the total pH scale and ΣH_2_S was calculated from H_2_S based on the pH values measured at the same depth. pH values are reported on the total scale.

### Flux Calculations

The sedimentary oxygen consumption was calculated from the oxygen depth profiles using Fick’s 1st law: $J_{o_{2}}=\phi \,\frac{D_{O_{2}}}{1-2\,\ln\left(\phi \right)}\,\frac{d\,\left[O_{2}\right]}{d\,x}$. Here, the diffusive oxygen flux (*J*_O_2_) across the sediment-water interface is calculated using the slope of the oxygen depth profile ($\frac{d\,\left[O_{2}\right]}{d\,x}$) and the molecular diffusion coefficient (*D*_O_2_) corrected for tortuosity (1−2*ln*⁡(ϕ)) and the mean porosity of the oxic zone (ϕ = 0.88). The molecular diffusion coefficient was calculated in R using the *marelac* package (Soetaert et al., 2010) taking into account the temperature (16°C) and salinity (30) of the incubations. Similarly the upward flux of ΣH_2_S was calculated using the slope of the ΣH_2_S depth profile using Fick’s first law at the base of the suboxic zone using the diffusion coefficient for H_2_S. Finally, upward fluxes were estimated from the iron pore water profiles using a similar approach and the molecular diffusion coefficient for ferrous iron (Fe^+2^). Flux data are presented as the mean ± SD with *n* = 4 for O_2_ and *n* = 2 for sulfide and ferrous iron fluxes.

### Sediment Sampling and DNA Extraction

On day 24, two cores per treatment were transferred into an anaerobic glove box (N_2_ atmosphere, Coy Laboratory Products, United States) and the sediment was sectioned at 0.5 cm resolution. Each slice of sediment was centrifuged at 4000 × *g* for 7.5 min, and the extracted pore water was collected and filtered through 0.22 μm cellulose acetate filters. Pore water samples were immediately stabilized with 50 μl ml^–1^ bidistilled HNO_3_ (65%, Suprapure, Merck) and stored for ∼3 weeks at 4°C until further analysis of Ca, Fe and Mn using ICP-OES (precision was <2% for all analytes).

The two remaining cores per treatment were sectioned in 0.5 cm thick sediment slices in ambient air. Per slice, the sediment was homogenized and subsamples were flash frozen in liquid N_2_ and stored at −80°C until DNA extraction. Another subsample was fixed with an equal volume of ethanol (Ethanol for molecular biology, Merck) and stored at −20°C for analysis by fluorescence *in situ* hybridization (FISH).

DNA was extracted from a weighed amount (∼0.5 g) of wet sediment following an amended protocol of Zhou et al. (1996) that is effective for cable bacteria lysis and isolating DNA from sediment. The sediment was incubated with lysozyme (final concentration 2.5 mg ml^–1^) and RNase (100 μg ml^–1^) in DNA extraction buffer (0.1 M Tris–HCl, 0.1 M sodium EDTA, 0.1 M sodium phosphate, 1.5 M NaCl, 1% hexadecyltrimethylammonium bromide and 1% poly[vinylpolypyrrolidone] (pH 8)) at 37°C for 30 min, followed by addition of SDS (to a final concentration of 0.5%) and proteinase K (final concentration 350 μg ml^–1^) and 30 min incubation at 37°C. Subsequently, the SDS concentration in the extraction liquid was increased to 1% and the incubation continued at 60°C for 2 h. Samples were mixed by inversion every 10–15 min during the whole incubation period. DNA contained in the aqueous phase was purified by two extractions with chloroform/isoamyl alcohol (24:1 v/v) and precipitated with 1 volume of 10% polyethylene glycol (molecular mass ∼ 8000 g/mol) overnight at 4°C. The precipitated DNA was collected by centrifugation, washed twice with ice-cold 70% ethanol and dissolved in 10 mM Tris–HCl (pH 8) buffer.

### qPCR Primers and Cycling Conditions

The abundance of (1) total bacteria, (2) bacteria belonging to the *Desulfobulbaceae* family, and (3) marine cable bacteria was estimated from the quantity of 16S rRNA gene copies determined by qPCR with target-specific primers (Table 1). Universal primers for total bacteria were Eub338 (Lane, 1991) and Eub518 (Muyzer et al., 1993). For quantification of *Desulfobulbaceae*, the forward primer DSBB280wF designed for the family *Desulfobulbaceae* was combined with the reverse primer SRB385R targeting sulfate-reducing bacteria (Amann et al., 1990; Sass et al., 1998). Primer DSBB280wF is an extension of primer DSBB280F targeting the family *Desulfobulbaceae* (Kjeldsen et al., 2007) allowing for a variation in the sequence (A instead of G at position 10) present in *Ca.* Electrothrix marina and *Ca.* E. aarhusiensis. For marine cable bacteria, forward primer ELF645wF was combined with reverse primer CB836wR. The ELF645wF primer is an extension of primer ELF645F targeting cable bacteria (Pfeffer et al., 2012; Marzocchi et al., 2014) to accommodate C instead of T at position 2 as present in the 16S rRNA gene sequence of *Ca.* E. marina. Reverse primer CB836wR is a newly designed primer that targets the genus *Ca.* Electrothrix, but also few Chloroflexi (see section “Results and Discussion”). Since this reverse primer is intended to be combined with forward primer ELF645wF it should have a similar melting temperature, in addition to the general requirements: GC content 40–60%, preferably start with G or C, no GC-rich end, no hairpin or self-dimer formation. The primer pair should be specific for cable bacteria and produce a relatively short amplicon (<200 bp) suitable for qPCR, and yield no hetero-dimer formation. A multiple alignment of the 16S rRNA gene sequence of cable bacteria (Trojan et al., 2016) and other *Desulfobulbaceae* (cf. Figure 4) was made with Muscle (Edgar, 2004) and manually inspected. Potential primer sequences specific for cable bacteria were identified and further assessed for specificity of the primer with Test Probe v 3.0, and specificity of the primer pair with TestPrime v 1.0, both against the Silva SSU reference database v 138 (Klindworth et al., 2013).

**TABLE 1:** Primers used for qPCR and for PCR-amplification of qPCR standards.

**Name**	**Sequence (5′-3′)^1^**	**Size (bp)**	**T_m_ (°C)^2^**	**References**
Eub338	ACT CCT ACG GGA GGC AGC AG	20	67.6	Lane, 1991
Eub518	ATT ACC GCG GCT GCT GG	17	65.1	Muyzer et al., 1993
DSBB280F	CGA TGG TTA GCG GGT CTG	18	62.1	Kjeldsen et al., 2007
DSBB280wF	CGA TGG TTA RCG GGT CTG	18	60.1–62.3	This study
SRB385R	CGG CGT CGC TGC GTC AGG	18	70.4	Amann et al., 1990; Sass et al., 1998
ELF645F	CTT GGC TTG AGT ATC AGA GG	20	59.9	Pfeffer et al., 2012; Marzocchi et al., 2014
ELF645wF	CYT GGC TTG AGT ATC AGA GG	20	60.1–61.8	This study
CB836R	CCT GCA CCT AGT TGA CAT CG	20	62.6	This study
CB836wR	CCT GCA YCT AGT TGA CAT CG	20	60.6–62.7	This study

*^1^ R = A/G, Y = C/T. ^2^Calculated using https://eu.idtdna.com/calc/analyzer with 3 mM MgCl_2_ and 0.2 μM primer concentration.*

qPCR analysis was performed with a realtime PCR analyzer (Bio-Rad CFX, Hercules, CA, United States and Rotor-Gene 6000, Corbett Research, Sydney, Australia) using ABsolute QPCR SYBR Green mix (Thermo Fisher scientific). The mix contained 3 mM MgCl_2_ and primer concentrations were 0.2 μM. Following hot start polymerase activation for 15 min at 95°C, the cycling conditions were: denaturation at 95°C for 15 s, annealing at 60°C (63°C for total bacteria) for 30 s, and extension at 72°C for 20 s. A series of 40 cycles was followed by 95°C for 30 s, then 60°C for 30 s and subsequently increasing the temperature to 95°C in steps of 0.5°C for 10 s/step, thus creating a melting curve for the obtained amplicons. Calibration curves were made using standards with at least 4 consecutive dilution steps (each step comprising a 10-time dilution). Sample DNA was analyzed in duplicate, and was diluted 10^4^ times for quantification of total bacteria and 10^2^ times for quantification of *Desulfobulbaceae* and cable bacteria. The detection limit of qPCR for *Desulfobulbaceae* and cable bacteria calculated using 10^2^ dilution of sample DNA and 10 copies μl^–1^ detected in a qPCR run was approximately 100 16S copies g^–1^ wet sediment.

### Standards for qPCR

Plasmid DNA with an inserted PCR-amplified partial 16S rRNA gene product was used to produce a calibration curve of 16S rRNA copy concentration vs. cycle quantification (C_q_) value with the *Desulfobulbaceae* and cable bacteria primer sets. DNA from the incubated sediment was PCR amplified with primers ELF645F-CB836R (for the cable bacteria standard, CB) at an annealing temperature (T_A_) of 60°C or with DSBB280F-SRB385R (for the *Desulfobulbaceae* standard, DSB) at T_A_ = 62°C using Taq DNA polymerase (New England Biolabs). Each PCR product was purified (EZNA Cycle Pure kit, Omega Bio-tek, Norcross, GA, United States) and ligated with the pCR^TM^ 4-TOPO TA vector and cloned into One Shot TOP10 Competent *E. coli* cells (Invitrogen) according to the manufacturer’s instructions. Insert-containing *E. coli* colonies were transferred to liquid medium, incubated overnight at 37°C and the cells harvested for plasmid isolation. For 20 clones each with DSB and CB inserts, the insert was PCR amplified using primers M13F and M13R and the PCR product sequenced (BaseClear, Leiden, Netherlands). This showed incorporation of an insert with the correct qPCR primer sites. Plasmid DNA was linearized by digestion with *Pst*I, purified and quantified by fluorescence (Qubit 3.0 and Qubit dsDNA HS assay kit; Life Technologies, Thermo Fisher Scientific).

For qPCR quantification of total bacteria, a PCR-amplified 16S rRNA gene product was used as the standard. DNA from incubated sediment (Lake Grevelingen) was amplified with general primers 27F (5′-AGA GTT TGA TCM TGG CTC AG-3′) and 1492R (5′-GGY TAC CTT GTT ACG ACT T-3′) using T_A_ = 55°C. The product was purified (EZNA Cycle Pure kit) and the DNA concentration quantified by absorbance at 260 nm (NanoDrop, Thermo Fisher scientific).

### Fluorescence *in situ* Hybridization

FISH analysis of cable bacteria using sediment from 0 to 0.5 cm depth was performed as described in Seitaj et al. (2015). One filter was prepared per sediment sample and the length of filaments hybridized to probe DSB706 was measured in 200 fields (105 × 141 μm).

### 16S rRNA Amplicon Sequence Analysis

The V4-V5 region of the 16S rRNA gene was amplified using DNA extracted from sediment with primers 515F-Y (5′-GTGYCAGCMGCCGCGGTAA-3′) and 926R (5′-CCGYCAATTYMTTTRAGTTT-3′) (Parada et al., 2016) with Illumina adapters added onto the target sequences. PCR was performed in triplicate using Q5 Hot start high-fidelity DNA polymerase (New England Biolabs). Cycling parameters were: initial denaturation at 98°C for 2 min, followed by 30 cycles of denaturation at 98°C for 10 s, annealing at 55°C for 20 s, extension at 72°C for 15 s, and final extension at 72°C for 2 min. Amplified DNA was checked for size on gel and triplicate products were pooled. Library preparation and sequencing (Illumina MiSeq, 2 × 300 bp) was performed at Eurofins Genomics, Ebersberg, Germany. The data for this study have been deposited in the European Nucleotide Archive (ENA) at EMBL-EBI under accession number PRJEB37045.

Amplicon sequence variants (ASVs) were analyzed using the R-package dada2 (Callahan et al., 2016). Only the forward reads, covering the V4 region of the 16S rRNA gene, were included in the analysis. The reads were filtered and chimeras were removed following the default settings of the dada2 pipeline. Singletons were ignored because of the higher chance that these originate from sequencing errors. The taxonomy of ASVs was assigned using the Silva small subunit rRNA nr database v132 (Quast et al., 2013).

### 16S Phylogeny

16S rRNA gene sequences of cable bacteria, other *Desulfobulbaceae* and sequences of clones from sediments were aligned with the software Muscle (Edgar, 2004) and manually inspected. Phylogenetic trees were calculated using maximum likelihood implemented in RAxML (Stamatakis, 2014) with Gamma rate heterogeneity and 1000 bootstraps. The short V4 amplicon sequences were added using the evolutionary placement algorithm, also implemented in RAxML.

### Filament Abundance of Cable Bacteria

The volumetric filament density N_F, volume_ (i.e., the length of cable bacterium filaments per cm^3^ of wet sediment) is calculated from the 16S copy density C_qPCR_ (i.e., the number of 16S copies per gram of wet sediment) determined by qPCR via the relation N_F, volume_ = C_qPCR_ × L_cell_ × ρ_w_/n_copy_, where L_cell_ is the mean length of an individual cell in a filament, ρ_w_ is the wet bulk density of the sediment (in g cm^–3^) and n_copy_ is the number of 16S rRNA gene copies present per cell. The aerial filament density N_F,area_ (i.e., the length of cable bacteria filaments per cm^2^ of sediment surface) is subsequently calculated as N_F,area_ = N_F,volume_ × L_CB_, where L_CB_ is the depth to which cable bacteria grow downward in the sediment.

## Results and Discussion

### qPCR Method Development: Primer Sets, Specificity and Calibration

Quantification of cable bacteria via qPCR requires an assessment of the performance of the primers that are used. Known cable bacteria sequences from surface sediments belong to two genera (Trojan et al., 2016): the genus *Ca.* Electrothrix encompassing the marine cable bacteria (4 species) and the genus *Ca.* Electronema, encompassing the freshwater cable bacteria (2 species). Ideal primer combinations for qPCR should allow annealing temperatures of 60°C or higher and generate a relatively small product (≤200 nucleotides). Based on these prerequisites, no suitable primer sequences were identified that could target both marine and freshwater cable bacteria with one single primer set. Therefore, we focused our quantification efforts on the genus *Ca.* Electrothrix that encompasses the marine cable bacteria.

The previously used primer ELF645F (Marzocchi et al., 2014) targets the genus *Ca.* Electrothrix, but for species *Ca.* E. marina, there is one mismatch at primer position 2. We allowed for this mismatch in the adapted primer ELF645wF. Analysis of primer ELF645wF using TestProbe showed that the primer targets only *Ca.* Electrothrix. When 1 mismatch is taken into account also other *Desulfobulbaceae* were targeted, with virtually all hits belonging to uncultured clones that were affiliated to the genus *Desulfobulbus*, comprising 9.2% of sequences in the *Desulfobulbus* lineage. We combined ELF645wF with a newly designed reverse primer CB836wR. Sequence CB836R matches to the species *Ca.* E. aarhusiensis, E. communis and E. japonica, and has 1 mismatch to *Ca.* E. marina that has been included in primer CB836wR. Analysis of CB836wR with TestProbe showed that it targets *Ca*. Electrothrix, and that there were also few perfect matches with uncultured clones affiliated to the order *Ardenticatenales* (Chloroflexi, 3 sequences) and to the genus *Desulfobulbus* (5 sequences). Primer CB836wR is a less specific primer compared to ELF645wF with in total 596 hits with 1 mismatch in the database, corresponding to sequences classified predominantly as *Desulfobulbaceae*, 55% of the lineage *Desulfobulbus*, but also Chloroflexi (7.4% of sequences affiliated to the order *Ardenticatenales*) and Gammaproteobacteria (10.2% of *Cardiobacterales*). The primer pair ELF645wF-CB836wR makes a more specific combination compared to the individual primers and is highly specific for *Ca*. Electrothrix. The primer combination does not target *Chloroflexi* and *Gammaproteobacteria* and, when allowing 1 mismatch for each primer, the only hits outside the *Ca*. Electrothrix lineage are 2 sequences affiliated to the lineage *Desulfobulbus* (out of 390 sequences affiliated to the genus *Desulfobulbus* in the database).

Members of the *Desulfobulbaceae* family were quantified using a specific primer targeting the family level [DSBB280F; (Kjeldsen et al., 2007)] that was extended to allow a mismatch with the 16S rRNA gene sequences of *Ca.* E. marina and E. aarhusiensis (DSBB280wF). The variant sequence had 31 hits in TestProbe, which were all affiliated to the *Desulfobulbales*. The forward primer DSBB280wF was combined with a more general primer for the class Deltaproteobacteria [SRB385R; (Sass et al., 1998)].

Quantitative PCR reactions using primer sets ELF645wF-CB936wR for cable bacteria (CB), DSB280wF-SRB385R for *Desulfobulbaceae* (DSB) and Eub338-Eub518 for total bacteria (TB) and the corresponding standards, all produced reproducible calibration curves with a calculated amplification efficiency of 92–104%, showing the suitability of the primer sets for qPCR-based enumeration. Hence, we used these primer sets for the quantification of CB, DSB, and TB in incubated marine sediment.

### Metabolic Activity of Cable Bacteria in Sediment Incubations

**FIGURE 1:**
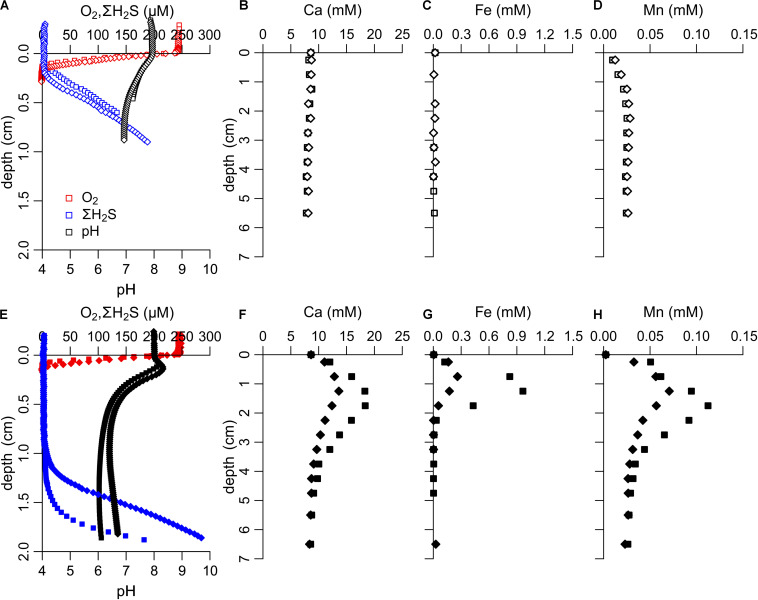
Pore water geochemistry of sediment in unamended incubations (upper row, open symbols) and stimulated incubations (bottom row, filled symbols). **(A,E)** Microsensor profiles of O_2_, ΣH_2_S and pH (*t* = 22 days). In the stimulated incubations a suboxic zone of ∼10 mm wide was present where both oxygen and sulfide were below detection limit, indicative of the activity of cable bacteria. **(B–D,F–H)** Pore water concentrations (*t* = 24 days) of Ca, Fe, and Mn were much higher in the stimulated compared to the unamended incubations, indicating dissolution of minerals stimulated by acidification of the suboxic zone through anodic sulfide oxidation by cable bacteria.

The metabolic activity of cable bacteria can be detected through their strong imprint on the pore water geochemistry, which can be verified by microsensor depth profiling (Meysman et al., 2015). Depth profiles of O_2_ and H_2_S obtained after 22 days of incubation showed that in the unamended incubation there was no development of a suboxic zone (i.e., a zone where both O_2_ and H_2_S are below detection limit) (Figure 1A), and no subsurface pH maximum occurred, thus indicating no detectable metabolic activity of cable bacteria. In the stimulated incubation, H_2_S was only detectable in deeper sediment layers, resulting in a suboxic zone that was ∼10 mm wide (Figure 1E). The oxygen penetration depth (1.01 ± 0.16 mm, *n* = 4) was also shallower compared to the unamended incubation (1.60 ± 0.25 mm, *n* = 4), indicating higher O_2_ consumption. Microsensor depth profiles of pH showed a subsurface peak coinciding with the O_2_ penetration depth (pH values 0.16–0.31 units above the pH of the overlying water, *n* = 2) and a decreased pH at depth (minimum pH values 6.0–6.4, at approximately 1.0–1.4 cm depth in the sediment, *n* = 2). This particular combination of O_2_, H_2_S, and pH depth profiles functions as the unique fingerprint of cable bacteria activity (Pfeffer et al., 2012; Meysman et al., 2015), and results from the spatial segregation of the anodic sulfide oxidation and cathodic oxygen reduction half-reactions in electrogenic sulfur oxidation.

In each treatment, pore water Ca, Fe and Mn was measured in the same cores as the microsensor depth profiles. Pore water concentrations of Ca, Fe and Mn showed a strong increase in the stimulated incubation compared to the unamended incubation (Figures 1B–D,F–H). Previous studies examining the effect of cable bacteria on sediment geochemistry have shown that the subsurface accumulation of Ca, Mn, and Fe is linked to the dissolution of Ca and Mn-carbonates and Fe sulfide. This mineral dissolution is a direct response to the acidification of the pore water (Figure 1E), resulting from proton production by the sulfide oxidation half-reaction performed by cable bacteria (Risgaard-Petersen et al., 2012; Rao et al., 2016). Together the microsensor depth profiles and the pore water data revealed a substantial metabolic activity of cable bacteria in the stimulated incubation and similar sets of depth profiles have been recorded in sediments with high cable bacteria activity (van de Velde et al., 2016; Malkin et al., 2017). No sign of cable bacteria activity was apparent in the unamended incubation.

**FIGURE 2:**
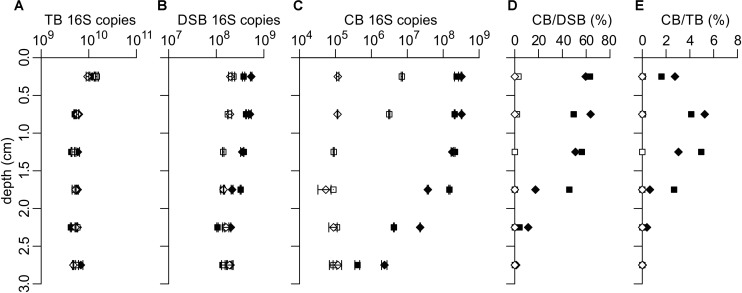
qPCR quantification of 16S rRNA gene copies of **(A)** total bacteria (TB), **(B)** *Desulfobulbaceae* (DSB) and **(C)** cable bacteria (CB). **(D)** CB 16S copies relative to DSB 16S copies and **(E)** CB 16S copies relative to TB 16S copies. Data is for two replicate cores each from unamended incubations (open symbols) and stimulated incubations (filled symbols). Data in panels **(A–C)** shows the mean with error bars denoting the standard deviation (*n* = 2). 16S rRNA gene copies are expressed per gram of wet sediment.

Together, these data suggest that the cable bacteria activity developed far more rapidly in the four sediment cores of the stimulated incubation compared to those of the unamended incubation (Figures 1, 2). In the stimulated incubation, “inoculum cores” were present that had already developed active cable bacteria, thus suggesting that a transfer occurred through the oxygenated overlying water. Such a transfer of cable bacteria via the water phase has not been documented previously, and the actual mechanism of transfer remains unclear. Yet, it suggests that cable bacteria can disperse through the water column and that they survive at least a short period in oxygenated water.

### qPCR Quantification of Bacterial Abundances

Quantification of cable bacteria, *Desulfobulbaceae* and total bacteria via qPCR was performed for each incubation treatment. The two replicate cores showed very similar abundances for all clades and treatments investigated, thus demonstrating good reproducibility of qPCR results (Figure 2). Total bacteria 16S rRNA gene copy numbers (“TB 16S copies”) were very similar in the unamended and stimulated incubations and were largest in the top 0–0.5 cm, 1.3 ± 0.2 × 10^10^ (g wet sediment)^–1^, compared to 5.4 ± 0.7 × 10^9^ (g wet sediment)^–1^ at >0.5 cm depth (Figure 2A). The increase of total bacteria in the top layer likely resulted from the growth of aerobic microorganisms at the sediment surface.

Quantification of *Desulfobulbaceae* in the unamended incubation showed a presence of approximately 1.5 ± 0.2 × 10^8^ 16S copies (g wet sediment)^–1^ below 1 cm depth (Figure 2B, open symbols). The DSB 16S copy number did not increase in the anoxic layers of the unamended incubation, indicating there was no enrichment of sulfate-reducing *Desulfobulbaceae* during the time of the incubation. In the stimulated incubation, the number of DSB 16S copies in the deepest layers (2–3 cm depth) was similar to the unamended incubation, but increased to 4.6 ± 0.8 × 10^8^ (g wet sediment)^–1^ in the 0–1 cm depth layer (Figure 2B, filled symbols). This increase in *Desulfobulbaceae* coincides with the suboxic zone and can be attributed to the presence of cable bacteria (see below).

The deeper layers (>1 cm) of the unamended incubation contained 9.1 ± 2.0 × 10^4^ cable bacteria 16S copies (g wet sediment)^–1^ (Figure 2C, open symbols), which we interpret to be the low background concentration in the homogenized sediments without an enriched and active population of cable bacteria. In one replicate of the unamended incubation, the CB 16S copy number remained constant throughout the depth profile, indicating cable bacteria had not developed. In the other replicate (core N1), CB 16S copies were 78 times higher in the upper 0–0.5 cm of the sediment [7.0 ± 0.3 × 10^6^ (g wet sediment)^–1^], an increase that corresponds to ∼6 doubling times. Assuming the growth rate is the same for all cells in a cable bacterium filament and using the doubling time of ∼20 h as reported in Schauer et al. (2014), this indicates that cable bacteria development likely started ∼5 days before core sectioning.

In the stimulated incubation, the number of detected CB 16S copies was 40–2400 times higher compared to the unamended incubation, amounting to 2.4–3.2 × 10^8^ 16S copies (g wet sediment)^–1^ in the upper 0.5 cm of sediment. The CB 16S copy numbers decreased to 0.4–2.3 × 10^6^ in the layer of 2.5–3 cm depth (Figure 2C, filled symbols). This confirms the accelerated development of cable bacteria in the stimulated incubation compared to the unamended incubation. The 16S CB copy number was constant within the suboxic zone (0–1.5 cm depth horizon), and rapidly declined below the depth at which free sulfide accumulated in the pore water (Figures 1E, 2C). A similar depth distribution has previously been observed in depth profiles of cable bacteria abundances quantified by FISH (Schauer et al., 2014; Vasquez-Cardenas et al., 2015; van de Velde et al., 2016).

Based on qPCR quantifications, CB 16S copies comprised up to 64% of DSB 16S copies in the stimulated incubation (Figure 2D). The difference between the DSB 16S copy numbers in the unamended and stimulated incubations matches the difference in CB 16S copy numbers between these treatments, demonstrating that the increase in the *Desulfobulbaceae* population is entirely due to the development of cable bacteria. Cable bacteria contributed up to 5.2% of the 16S copies of the total bacterial population within the suboxic zone (depth 0.5–1.5 cm), while a lower contribution of 1.6–2.7% is seen in the oxic zone (0–0.5 cm layer) due to the larger number of total bacteria in the oxic surface layer (Figures 2A,E). Studies of the microbial community composition of marine environments using 16S amplicon sequencing have found cable bacteria abundances up to 0.6% in Baltic Sea sediments (Klier et al., 2018), and up to 4.5% in sediment from a shallow lagoon in Aarhus Bay (Klier et al., 2018; Otte et al., 2018). The largest relative abundance at the Aarhus Bay site was detected in sediment sampled at 3–10 mm depth, consistent with the results here.

The observed abundance data illustrate once more that cable bacteria can grow to dense filament networks in aquatic sediments (Schauer et al., 2014; Vasquez-Cardenas et al., 2015; Malkin et al., 2017). If we assume that individual cells are L_cell_ = 3 μm long (based on FISH imaging, cf. Figure 5), and there are two 16S rRNA gene copies present per cell [as for the genome sequence of *Ca*. Electrothrix aarhusiensis; (Kjeldsen et al., 2019)], and sediment wet bulk density is ρ_w_ = 1.32 g cm^–3^ sediment, then our observed cable bacteria abundance of C_qPCR_ = 3 × 10^8^ 16S copies (g wet sediment)^–1^ corresponds to a volumetric filament density of 600 m per bulk cm^3^ of sediment. This filament length density is at the high end of densities obtained with FISH under field conditions, which are in the range of 2–290 m per cm^3^ bulk sediment (Malkin et al., 2014; Burdorf et al., 2016; van de Velde et al., 2016; Marzocchi et al., 2018). However, in laboratory sediment incubations, similar to the ones conducted here, filament lengths of cable bacteria can amount up to 2800 m per cm^3^ bulk sediment (Schauer et al., 2014; Vasquez-Cardenas et al., 2015; Malkin et al., 2017).

After integration with depth, assuming that cable bacteria filaments extend to a depth of 1.5 cm (as derived from the qPCR data in Figure 2C), we obtain an aerial filament density of 900 m per cm^2^ of sediment surface area. This is again larger than FISH based values under field conditions, which range up to 480 m per cm^2^ (Risgaard-Petersen et al., 2015; Seitaj et al., 2015; Burdorf et al., 2016; van de Velde et al., 2016; Marzocchi et al., 2018; Hermans et al., 2019). Assuming that all filaments are straight and vertically oriented in the sediment (i.e., filaments are 1.5 cm long), we find an areal density of 6 × 10^4^ filaments cm^–2^, which provides an average horizontal distance of 45 μm between filaments.

### Cable Bacteria and *Desulfobulbaceae* Abundance Assessed by Amplicon Sequencing

Amplicon sequencing of the V4 region of the 16S rRNA gene yielded ≥52,000 sequences per sample (Supplementary Figure S2 shows the microbial community composition). Using the qPCR TB 16S copy number per core and depth layer, the calculated detection limit for the amplicon sequencing variant (ASV) abundance ranged between 5 × 10^4^ and 4 × 10^5^ 16S copies (g wet sediment)^–1^. The ASV detection limit was in the same range as the qPCR CB 16S copy number in sediment layers where cable bacteria were not enriched during incubation, and therefore, amplicon sequencing could not be used to accurately assess the abundance of cable bacteria in most depth layers of the unamended incubation.

**FIGURE 3:**
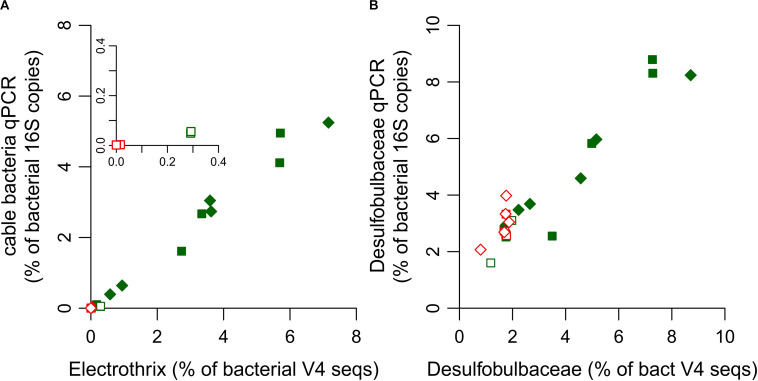
Ratio of the relative abundance of **(A)** cable bacteria and **(B)** *Desulfobulbaceae* quantified by qPCR and by classification of amplicon sequences. For panel **(A)**, relative abundance of cable bacteria by qPCR (*y*-axis) was calculated as CB 16S copies/TB 16 copies (%), and relative abundance by amplicon sequencing (*x*-axis) as Σreads classified as *Ca*. Electrothrix/Σreads classified as Bacteria (%). For panel **(B)**, calculations were similar, using DSB 16S copies, and reads classified as *Desulfobulbaceae*. Each data point shows the ratio for one depth layer in one of the replicate cores of the unamended incubation (open symbols) and stimulated incubation (filled symbols). The inset in panel **(A)** shows a close-up for unamended incubation N1. Ratios for depth layers with cable bacteria present are shown in green, and for depth layers without cable bacteria in red.

Amplicon sequences related to *Ca.* Electrothrix were detected, but no sequences were related to *Ca.* Electronema, consistent with the known ecology of the two genera. *Ca.* Electrothrix is found in marine environments, similar to the incubations here, while *Ca.* Electronema occurs in freshwater environments (Trojan et al., 2016). The abundance of cable bacteria relative to total bacteria assessed by qPCR and amplicon sequencing corresponded well for the sediment layers in the stimulated incubation (slope = 0.77, *r*^2^ = 0.99; Figure 3A). However, there was one notable exception. In core N1 of the unamended incubation, qPCR detected only ∼20% of the amplicon sequences that were assigned to *Ca*. Electrothrix (Figure 3A inset), which was caused by primer mismatch (as discussed below). In core N2 of the unamended incubation, no Electrothrix related sequences were detected, in agreement with qPCR results.

The relative abundance of *Desulfobulbaceae* estimated by classification of amplicon sequences and qPCR showed discrepancies in sediment layers without cable bacteria (Figure 3B). In layers without cable bacteria, qPCR overestimated *Desulfobulbaceae* by approximately 70% (slope = 1.72, *r*^2^ = 0.14), while in sediment layers with cable bacteria, qPCR and amplicon sequencing data showed good agreement (slope = 1.10, *r*^2^ = 0.89). It is likely that PCR amplification of other sequences than *Desulfobulbaceae* caused this overestimation. Since the sequenced V4 region is outside the region targeted by the DSB primers, we cannot evaluate if sequences that were classified as *Desulfobulbaceae* have mismatches with the primers used for qPCR. A TestProbe query of the DSBB280wF primer shows that there are a few thousand sequences with 1 mismatch present in the database that are affiliated to other families in the Deltaproteobacteria, and which hence may anneal with the reverse primer SRB385R. The SRB385R primer itself has >100,000 hits in the database. The use of a reverse primer that is (more) specific for the *Desulfobulbaceae* could likely decrease the incidence of misidentification.

### Metabolic Activity of Cable Bacteria at the Single Filament and Single Cell Level

The convergence of amplicon sequencing and qPCR estimates suggests we can attain reliable estimates of densities of cells and filaments of cable bacteria in our sediment incubations. If we combine these data with estimates of the rate of long-distance electron transport, we can obtain insight into the electron flow at the single filament and single cell level. The electrical current density due to cable bacteria activity can be derived from either the rate of cathodic oxygen reduction or the rate of anodic sulfide oxidation (Meysman et al., 2015; van de Velde et al., 2016). Both rates can be estimated from the geochemical flux data (Figures 1, 2), and in theory, the resulting value for the current density should be the same (Meysman et al., 2015).

The sedimentary oxygen consumption was 35.1 ± 7.7 mmol O_2_ m^–2^ d^–1^ in the stimulated incubations with cable bacteria and 21.6 ± 3.7 mmol O_2_ m^–2^ d^–1^ in the unamended incubations with a low density of cable bacteria. The difference between both, 13.6 ± 11.4 mmol O_2_ m^–2^ d^–1^ or 39% of the total O_2_ uptake, can be attributed to cathodic oxygen reduction by cable bacteria, matching previous field studies, which estimate that 5–50% of total O_2_ uptake can be attributed to e-SOx (Malkin et al., 2014; van de Velde et al., 2016). Knowing that 4 electrons are transferred to each O_2_ molecule during cathodic O_2_ reduction, this provides a current density of 61 ± 51 mA m^–2^ (1A = 1.036 × 10^–5^ mol e^–^ s^–1^). Current densities reported for different field studies range from 3.8 to 47 mA m^–2^ (Malkin et al., 2014, 2017; van de Velde et al., 2016, 2017) and go up to 74–96 mA m^–2^ for sediment incubations under laboratory conditions (Risgaard-Petersen et al., 2012; Schauer et al., 2014; Rao et al., 2016; van de Velde et al., 2017). Accordingly, our observed current density is at the high end of the field observations, and at the low end of the laboratory experiments.

A sulfide balance for the stimulated incubation allows a second estimate of the current density. There is a sulfide flux of 4.8 ± 1.3 mmol H_2_S m^–2^ d^–1^ from the deeper sulfidic zone toward the suboxic zone (Figure 1E), which is generated by sulfate reduction in deeper sediment (sediment zone = 59 mm wide). If we assume that the volumetric rate of sulfate reduction is similar in the suboxic zone (sediment zone = 10 mm wide), we can estimate that sulfate reduction produces 0.8 ± 0.2 [ = (10/59) × 4.8] mmol H_2_S m^–2^ d^–1^ in the suboxic zone. Anodic sulfide oxidation is the sum of consumption of sulfide generated by sulfate reduction in the suboxic zone (0.8 mmol H_2_S m^–2^ d^–1^), sulfide generated by sulfate reduction in the deeper sediment (4.8 mmol H_2_S m^–2^ d^–1^) and sulfide generated by net FeS dissolution (−2.0 ± 1.3 mmol H_2_S m^–2^ d^–1^ derived from the upward flux of dissolved Fe; the downward Fe flux is not accounted for as we assume this precipitates as FeS). Anodic oxidation of sulfide (total rate 7.6 mmol H_2_S m^–2^ d^–1^) produces 8 electrons when sulfide is oxidized to sulfate, and hence, the current density is estimated at 68 ± 25 mA m^–2^, which closely matches our estimate based on cathodic O_2_ consumption.

Based on the above values for the current density (61–68 mA m^–2^) and the aerial cable bacteria density (6 × 10^8^ filaments m^–2^), we can calculate the metabolic activity of an average individual cable bacterium filament as 100−115 pA per filament. When cable bacteria were connected directly with electrodes with an applied voltage bias of 100 mV, measured currents ranged from 6 to 1350 pA per cable bacterium filament (Meysman et al., 2019). It should be noted, however, that a direct comparison is difficult, as the distance over which currents were transmitted was different. Nevertheless, the magnitude of currents observed under *in situ* conditions matches that of previous *in silico* electrical measurements.

The pathway by which oxygen is reduced in cable bacteria is currently unknown. Recent ^13^C and ^15^N labeling studies have shown that cells in the oxic zone of the sediment show very little or no carbon or nitrogen assimilation, implying that oxygen reduction in cable bacteria is not coupled to energy conservation (Geerlings et al., 2020). Moreover, no terminal oxidases could be identified in cable bacteria genomes and it was hypothesized that cable bacteria reduce oxygen via periplasmic cytochromes (Kjeldsen et al., 2019).

The cell specific oxygen consumption rate of cable bacteria in the oxic zone calculated from the cathodic oxygen consumption, the oxygen penetration depth (∼1 mm; Figure 1E) and qPCR enumerated cable bacteria is 69 ± 58 fmol O_2_ cell^–1^ day^–1^, which is within the same order of magnitude as the value of 36 fmol O_2_ cell^–1^ day^–1^ reported by Schauer et al. (2014). Measurements of the oxygen reduction capacity of cable bacteria using cyclic voltammetry resulted in a rate of 6.1 pmol O_2_ cell^–1^ day^–1^ (at 50 μM O_2_; Geerlings et al., 2020), hence the potential capacity for oxygen reduction is approximately a hundred times larger than the actual rates in sediment incubations.

### Cable Bacteria Diversity and qPCR Quantification

**FIGURE 4:**
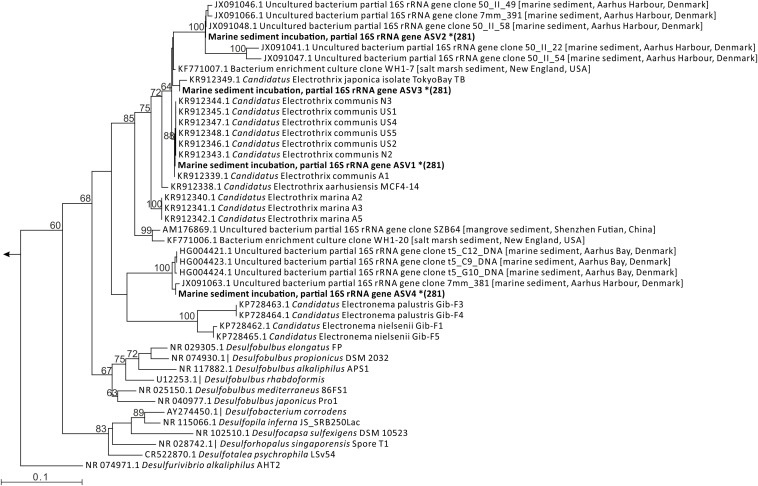
Phylogenetic tree of 16S rRNA gene sequences of cable bacteria, cultured members of the *Desulfobulbaceae* and selected clones from sediment (the origin of the sediments is indicated in square brackets) and the amplicon sequence variants (ASVs) classified as *Ca.* Electrothrix detected in the incubations of marine sediment (in bold). The ASV sequences (indicated with * followed by the length of the sequence) were inserted using the evolutionary placement algorithm. Bootstrap values (%) were calculated for 1000 re-samplings; only values ≥60 are shown.

Up to present, four different candidate species (communis, aarhusiensis, japonica and marina) have been identified within the marine cable bacteria genus *Ca.* Electrothrix (Trojan et al., 2016), and field research shows that these different species of cable bacteria may co-exist within the same sediment environment (Marzocchi et al., 2018). In the unamended and stimulated incubations together, four amplicon sequence variants (ASV1-ASV4) were identified that classified as *Ca.* Electrothrix. ASV1 showed 100% sequence identity to both *Ca.* E. communis and E. aarhusiensis, ASV2 was most closely related to E. marina with 97.2% identity, ASV3 shared 100% sequence identity with E. japonica, and ASV4 shared 97.2% sequence identity with both E. communis and E. aarhusiensis. Placement of the ASVs in a phylogenetic tree of 16S rRNA sequences of cable bacteria, other *Desulfobulbaceae* and sediment clones (Figure 4) showed that ASV1 and ASV3 clustered with the currently described species of marine cable bacteria. Placement of ASV1 with *Ca*. E. communis and E. aarhusiensis is equally likely, since the sequences have identical V4 regions. ASV2 was placed with a group of clones that clustered more distant from the described *Ca.* Electrothrix species, but still in the Electrothrix lineage. ASV4 was placed with another group of sediment clones, which clustered outside of this lineage, however, with low bootstrap support. The sediment clone sequences included in the tree were obtained from sediment incubations with cable bacteria, using sediment from Aarhus Bay, Denmark (Pfeffer et al., 2012; Schauer et al., 2014). Sequence identity between ASV2 and ASV4 and the related clones is 99–100%.

Quantitative PCR is a relatively rapid and low-cost method for quantification of microbial populations, but its success depends inherently on the conservation of primer sites in the targeted populations. The sequenced V4 region contains the ELF645wF primer site, which showed full sequence identity for ASV1 and ASV3, but two mismatches for ASV4 (positions 1 and 3) and three mismatches for ASV2 (positions 2, 3 and 5). The ASV1 contributed up to 7.0% of bacterial V4 sequences, and ASV3 up to 0.62%. The ASVs 2 and 4 made up small proportions of sequences classified as Electrothrix in sediments of the stimulated incubation. However, in core N1 from the unamended incubation, ASV2 was the only variant detected. The presence of sequence mismatches in the ELF645wF primer site of ASV2 explains the underestimation of cable bacteria by qPCR in incubation N1 (Figure 3A).

**FIGURE 5:**
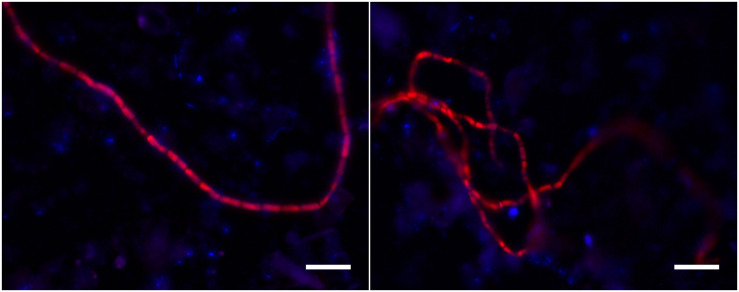
FISH images of filamentous bacteria hybridized with DSB706 from sediment of core N1 of the unamended incubation. The images show the overlay of false colored images for the DSB706 (red) and DAPI (blue) signal. The scale bars denote 10 μm.

So the question arises whether the ASVs 2 and 4 are indeed cable bacteria? FISH analysis of sediment from the N1 incubation (which only has ASV2) showed hybridization of filamentous bacteria to probe DSB706, supporting that ASV2 belongs to cable bacteria (Figure 5). The placement of sequence ASV2 in the cable bacteria lineage in the 16S rRNA phylogenetic tree (Figure 4) also supports that this ASV is representative of a cable bacterium strain. For sequence ASV4 and also clones previously obtained from marine sediment incubations (Figure 4; Pfeffer et al., 2012; Schauer et al., 2014), it is yet unclear if they belong to cable bacteria.

For sediment incubations with nitrate, Marzocchi et al. (2014) observed that of bacterial filaments that hybridized with probe DSB706, only 21 ± 13% also hybridized with probe ELF645. FISH probe ELF645 targets the same sequence as primer ELF645F. Hence, based on the observations of Marzocchi et al. (2014), the diversity of marine cable bacteria appears larger than currently identified, which could lead to an underestimation of qPCR-based abundance data of cable bacteria. Therefore, a combination of different primers targeting different subgroups of cable bacteria may be needed for qPCR quantification, or primers for other targets, e.g., the 23S rRNA sequence, or genes with a function in the metabolism of cable bacteria.

## Data Availability Statement

The datasets presented in this study can be found in online repositories. The names of the repository/repositories and accession number(s) can be found in the article.

## Author Contributions

JG and FM designed the study. JG designed the qPCR primers, performed qPCR analysis, and analyzed the amplicon sequencing data. SV performed the sediment incubations and the pore water analysis. All authors contributed to the article and approved the submitted version.

## Conflict of Interest

The authors declare that the research was conducted in the absence of any commercial or financial relationships that could be construed as a potential conflict of interest.
