# Supplementary material for: Quantification of Cable Bacteria in Marine Sediments via qPCR
Source: Front Microbiol. 2020 Jul 3;11:1506. doi: 10.3389/fmicb.2020.01506 (PMC7348212; doi:10.3389/fmicb.2020.01506)
Supplement: Supplementary file 1 [file Data_Sheet_1.PDF]

Supplementary Material

Quantification of cable bacteria in marine sediments via qPCR

Jeanine S. Geelhoed[1*], Sebastiaan J. van de Velde[2], Filip J.R. Meysman[1,3*]

[1]Department of Biology, University of Antwerp, Antwerpen, Belgium

[2] Department of Earth and Planetary Sciences, University of California Riverside, Riverside, CA, USA

[3] Department of Biotechnology, Delft University of Technology, Delft, The Netherlands

1 Supplementary Figures and Tables

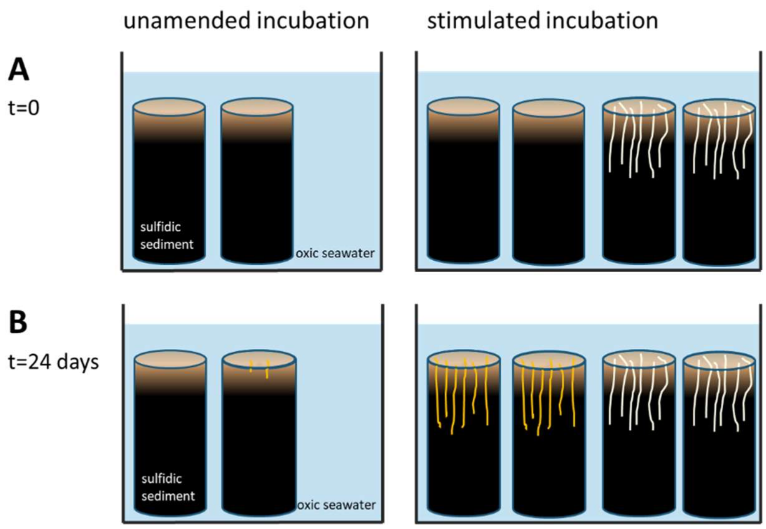

Supplementary Figure 1. Schematic drawing of the experimental setup. Only 2 of the 4 cores per treatment are shown. Homogenized and sieved sediment was packed into core liners and incubated in overlying oxygenated seawater. (A) Experimental setup at the start of the incubation (t=0): 'unamended' incubation of sediment cores in overlying freshly prepared artificial seawater, 'stimulated' incubation of sediment cores together with sediment cores of which incubation was started 32 days earlier and that already contained active cable bacteria. (B) Situation after 24 days of incubation: 'unamended' incubation sediment cores contain no or very few cable bacteria, 'stimulated' incubation sediment cores contain more cable bacteria that also extend deeper into the sediment.

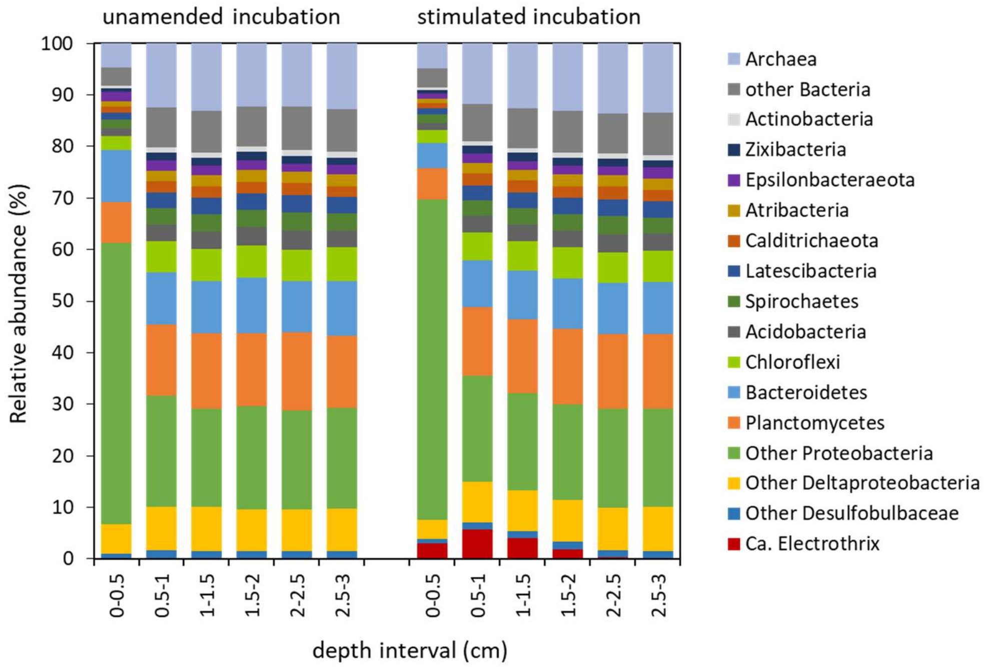

Supplementary Figure 2. Microbial community composition of the unamended incubation and the stimulated incubation after 24 days of incubation (average, n=2). Shown is the relative abundance of V4 amplicon reads classified as *Ca*. Electrothrix, other *Desulfobulbaceae* than *Ca*. Electrothrix, other *Deltaproteobacteria* than *Desulfobulbaceae*, other Proteobacteria than *Deltaproteobacteria*, and other major Phyla. Reads were classified against the Silva nr database v132.
